# Author Correction: Inducible expression of large gRNA arrays for multiplexed CRISPRai applications

**DOI:** 10.1038/s41467-023-35867-9

**Published:** 2023-01-10

**Authors:** William M. Shaw, Lucie Studená, Kyler Roy, Piotr Hapeta, Nicholas S. McCarty, Alicia E. Graham, Tom Ellis, Rodrigo Ledesma-Amaro

**Affiliations:** 1 Imperial College Centre for Synthetic Biology, Imperial College London, London, SW7 2AZ UK; 2 Department of Bioengineering, Imperial College London, London, SW7 2AZ UK

**Keywords:** Metabolic engineering, Metabolic engineering, Synthetic biology, CRISPR-Cas9 genome editing

Correction to: *Nature Communications* 10.1038/s41467-022-32603-7, published online 25 August 2022

The original version of the Supplementary Information associated with this Article did not include links to the Benchling sequences indicated in “For an example assembly using the gRNAs from Fig. 2a, please follow the Benchling link here” in the legend of Supplementary Figure 3 and in Supplementary Table 1. The HTML has been updated to include a corrected version of the Supplementary Information.

## Supplementary information

Updated Supplementary Information

