# Supplementary material for: Inducible expression of large gRNA arrays for multiplexed CRISPRai applications
Source: Nat Commun. 2022 Aug 25;13:4984. doi: 10.1038/s41467-022-32603-7 (PMC9411621; doi:10.1038/s41467-022-32603-7)

Supplementary Information for

Inducible expression of large gRNA arrays for multiplexed CRISPRai applications

William M. Shaw[1,2,3], Lucie Studená[1,2,3], Kyler Roy[1,2], Piotr Hapeta[1,2], Nicholas S. McCarty[1,2], Alicia E. Graham[1,2], Tom Ellis[1,2] and Rodrigo Ledesma-Amaro[1,2,*]

[1]Imperial College Centre for Synthetic Biology, Imperial College London, London, SW7 2AZ, UK,
[2]Department of Bioengineering, Imperial College London, London, SW7 2AZ, UK,
[3]These authors contributed equally: William M. Shaw, Lucie Studená
[*]Corresponding author: Rodrigo Ledesma-Amaro r.ledesma-amaro@imperial.ac.uk

This Supplementary Information includes:

Supplementary Fig. 1. Expression of GFP as a proxy for basal gRNA array transcription.

Supplementary Fig. 2. Limits of gRNA array silencing from mutTetR in the uninduced state.

Supplementary Fig. 3. gRNA array fragment generation and assembly.

Supplementary Fig. 4. Assembly of sub-arrays and spacers into the CRISPRai vector.

Supplementary Fig. 5. Stability of CRISPRai in batch culture.

Supplementary Fig. 6. Stability of CRISPRai over 1 week of daily cell passaging.

Supplementary Table 1. Inducible CRISPRai toolkit plasmids.

Supplementary Table 2. gRNA targets used in this study.

Gating strategy for flow cytometry.

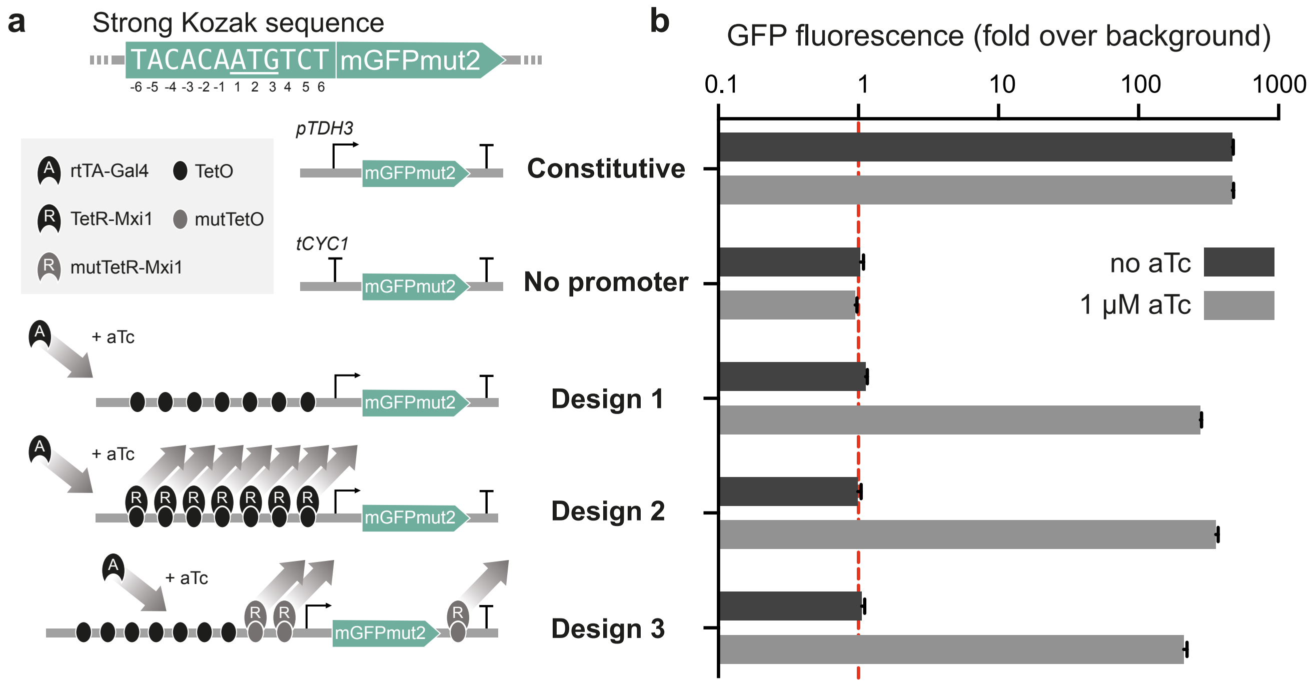

Supplementary Fig. 1. Expression of GFP as a proxy for basal gRNA array transcription. a, The gRNA array of the five designs in Figure 1 were precisely replaced with the coding region of mGFPmut2, prepended with a strong Kozak sequence. **b**, GFP fluorescence measurements of the five expression systems in the presence and absence of 1 µM aTc. Measurements were divided by a no-GFP control (wildtype BY4741 yeast) to determine basal expression of GFP. 470-fold expression of GFP was seen in the Constitutive yeast (using the strong *TDH3* promoter) validating substitution of the gRNA array with the Kozak-mGFPmut2 coding sequence leads to protein expression. No significant difference was seen between the control yeast and No promoter yeast in both the presence and absence of aTc, demonstrating the *CYC1* terminator does not confer cryptic promoter activity or transcriptional read-through from upstream expression. No significant difference was seen between the control yeast and Design 1-3 yeast, in line with previous experiments that revealed tight control of transcription initiation at the designed transcription start site in the uninduced state. High fold change in expression was seen in Design 1-3 in the presence of inducer, as expected. Experimental measurements are mGFPmut2 fluorescence levels per cell as determined by flow cytometry and shown as the mean ± SD from eight biological replicates.

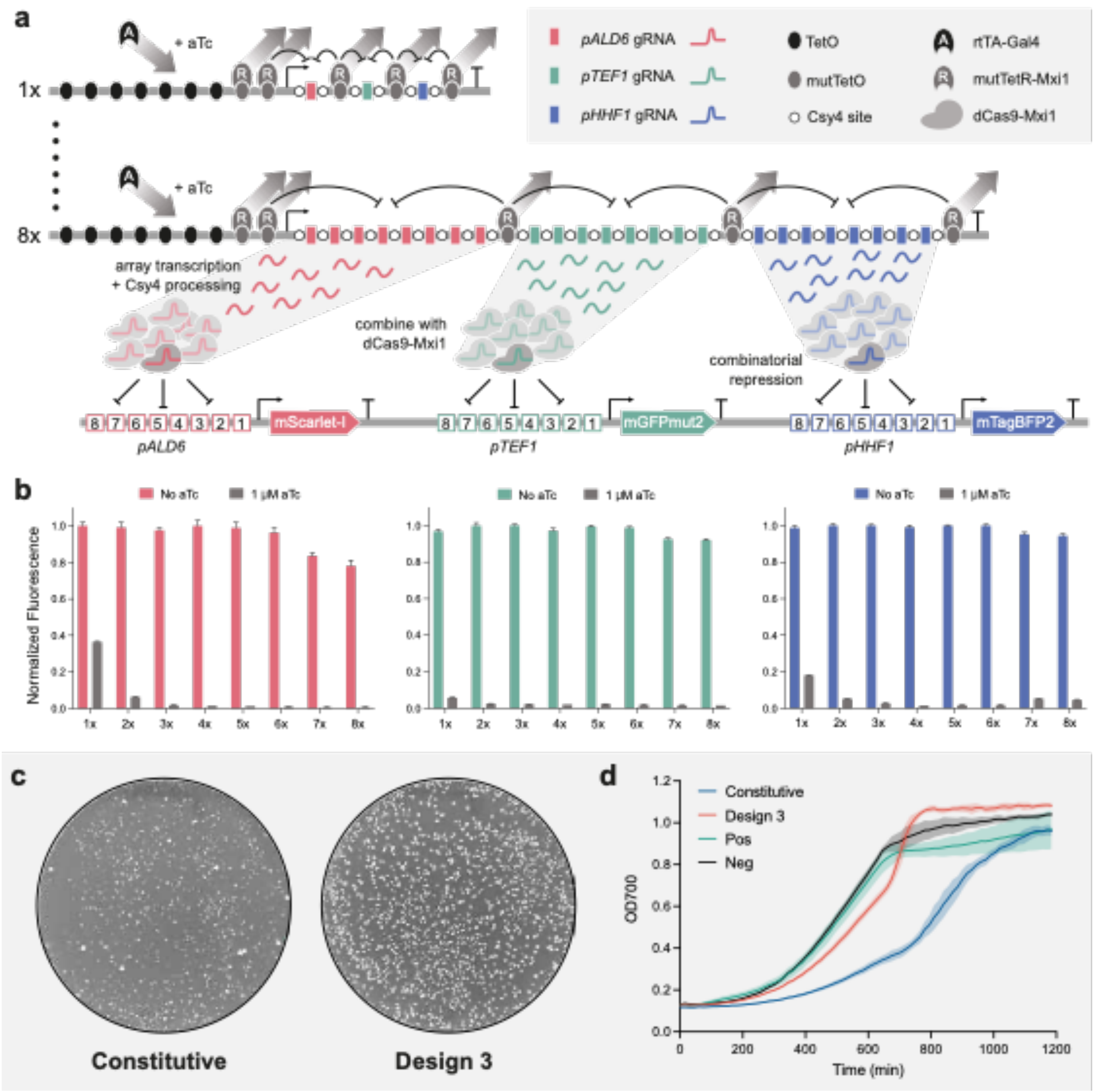

Supplementary Fig. 2. Limits of gRNA array silencing from mutTetR in the uninduced state. a, mutTetR silencing of the array with mutTetO sites either side of groups of 1-8 gRNAs targeting the *ALD6*, *TEF1*, and *HHF1* promoters driving the expression of mScarlet-I, mGFPmut2, mTagBFP2, respectively. Expression of the array followed by Csy4 processing and dCas9-Mxi1 mediated repression of the target promoters. **b**, Fluorescence measurements of the inducible gRNA systems combinatorially repressing *pALD6*-mScarlet-I, *pTEF1*-mGFPmut2, and *pHHF1*-mTagBFP2, in the presence and absence of 1 µM aTc, normalised to a no-gRNA and a no-fluorescent protein control. Experimental measurements are mScarlet-I, mGFPmut2, and mTagBFP2 fluorescence levels per cell as determined by flow cytometry and shown as the mean ± SD from four biological replicates. **c**, Transformation of 6x Constitutive array (left) and 6x Design 3 array (right) without inducer. Larger colonies in the Constitutive condition are escape mutants that have deleted various regions of the gRNA array, as confirmed by colony PCR (data not shown). **d**, Growth curves of Constitutive and Design 3 yeast in the absence of inducer, compared to yeast cells with only fluorescent protein reporters (Pos) and wildtype BY4741 yeast (Neg). Experimental measurements are optical density at OD_{700} as measured in a plate reader and shown as the mean (line) ± SD (shaded) from six biological replicates.

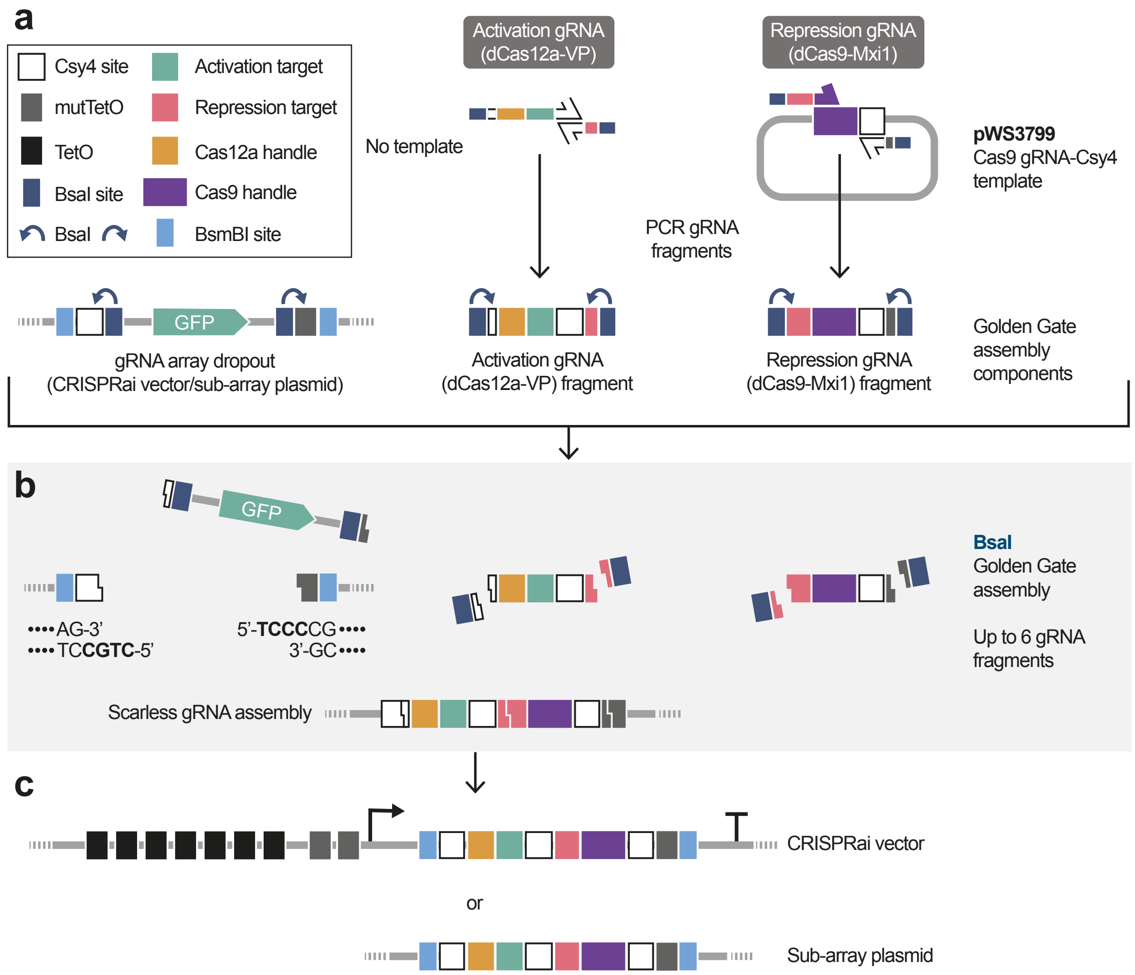

Supplementary Fig. 3. gRNA array fragment generation and assembly. a, Example PCR of gRNA fragments for a 2x gRNA array containing an activation (dCas12a-VP) and repression (dCas9-Mxi1) guide. Activation gRNAs are designed to include the entire gRNA fragment sequence (Cas12a handle (AATTTCTACTAAGTGTAGAT), 20 bp target sequence, Cys4 site, and flanking BsaI cloning sequences) within the primers. The primers are designed to anneal to each other at the Csy4 site and use 5 rounds of PCR extension, without a template, to complete the full dsDNA gRNA fragment. Repression gRNAs are designed to include the 20 bp target sequence, flanking BsaI cloning sequences, and Cas9 handle and Csy4 site priming sequences within the primers, which then amplify the full Cas9 handle and csy4 site from the pWS3799 template plasmid to create the full dsDNA gRNA fragment after 30 cycles of PCR. The BsaI-generated overhangs in the CRISPRai vector and sub-array plasmid are within the last (GCAG) and first (TCCC) 4 bp of the Csy4 and mutTetO sites, respectively. By designing the BsaI-generated overhangs between gRNAs to occur within the flanking sequence of the gRNA fragment, such as the 20 bp targeting sequence, gRNAs within the array can be assembled scarlessly to be precisely flanked by Csy4 sites. **b**, gRNA fragments are purified and included in a BsaI Golden Gate assembly reaction with the CRISPRai vector or sub-array plasmid. Completed reactions are transformed into *E. coli* and initially screened for the absence of green fluorescence on a blue light box (assembled gRNA arrays replace the *E. coli* GFP expression cassette dropout in the backbone). Correct array identity is then confirmed by colony PCR and Sanger sequencing across the entire array following DNA plasmid isolation by miniprep. **c**, Assembled gRNA arrays in the CRISPRai vector and sub-array plasmid. Fully assembled arrays in the CRISPRai vector position mutTetO sites either side of the gRNA cluster for efficient silencing. Sub-arrays position a single mutTetO site downstream of the sub-array. Assembly of sub-arrays into the CRISPRai vector results in all sub-arrays flanked by mutTetO sites for efficient silencing (**Supplementary Fig. 5**). Plasmid backbones not shown.

For an example assembly using the gRNAs from Fig. 2a, please follow the Benchling link here.

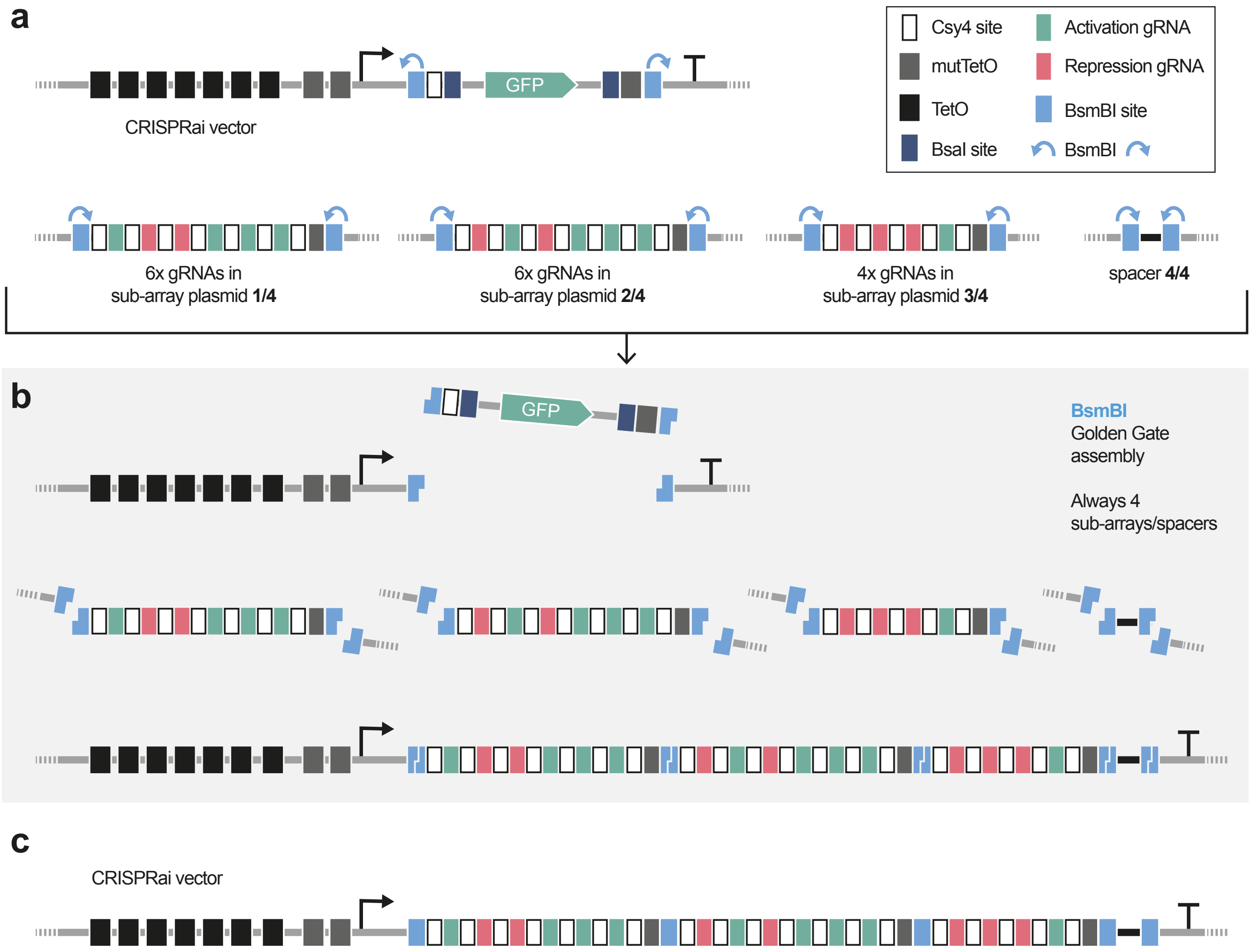

Supplementary Fig. 4. Assembly of sub-arrays and spacers into the CRISPRai vector. Example assembly of a 16x gRNA array from 3 pre-assembled sub-arrays and a 50 bp spacer into a CRISPRai vector by BsmBI Golden Gate assembly. All assemblies from sub-arrays and spacers comprise 4 sub-array/spacers with the appropriate BsmBI overhangs (1/4, 2/4, 3/4, and 4/4). Sub-arrays and spacers are flanked by pre-defined BsmBI-generated overhangs that organise their position within the array during the BsmBI Golden Gate assembly. **b**, BsmBI assembly of 3 sub-arrays and a spacer into the CRISPRai vector. As with gRNA fragment assembly, completed reactions are transformed into *E. coli* and screened for the absence of green fluorescence on a blue light box. Correct array identity is confirmed by colony PCR or restriction digest using unique restriction enzyme sites either side of the entire array (Left; EcoRI/XbaI, Right; SpeI/PstI) following DNA plasmid isolation by miniprep. **c**, Fully assembled arrays position mutTetO sites either side of each sub-array. Efficient silencing is seen up to 6 gRNAs in each sub-array. Five 4 bp BsmBI cloning scars result from sub-array assembly (blue). However, these are positioned outside the Csy4 sites and so are not included in the mature gRNA, keeping the Csy4 processed gRNAs within the array free of additional RNA sequence (excluding the cleaved Csy4 sequence). Spacers are 50 bp of biologically neutral DNA designed by R2oDNA designer (Casini et al. (2014) DOI: 10.1021/sb4001323).

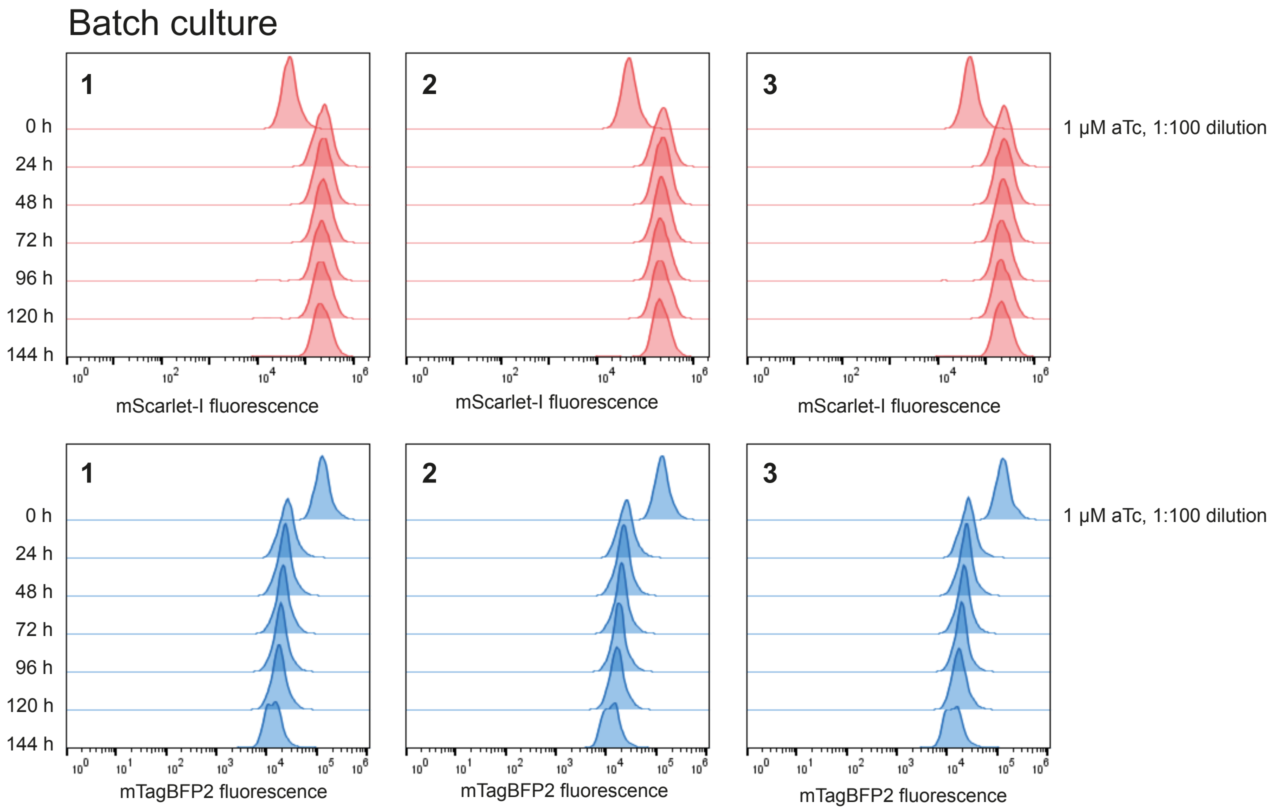

Supplementary Fig. 5. Stability of CRISPRai in batch culture. Histograms of mScarlet-I (Top) and mTagBFP2 (Bottom) fluorescence data from **Fig. 3b**. Saturated overnight cultures were diluted 1:100 into fresh SC media with 1 μM aTc at 0 h and growth at 30 °C shaking. Cells were sampled at 24 h increments with no further changes to initial conditions. After 24 h, mScarlet-I and mTagBFP2 had reached maximum and minimum fluorescence levels, respectively. Cell fluorescence levels were maintained over the course of 6 days measurements were collected, with cell populations remaining as a single peak, suggesting no observable CRISPRai escape mutants. Experimental measurements are mScarlet-I (red) and mTagBFP2 (blue) fluorescence per cell as determined by flow cytometry and shown as histograms of all gated events from individual biological replicates (> 10,000 cells).

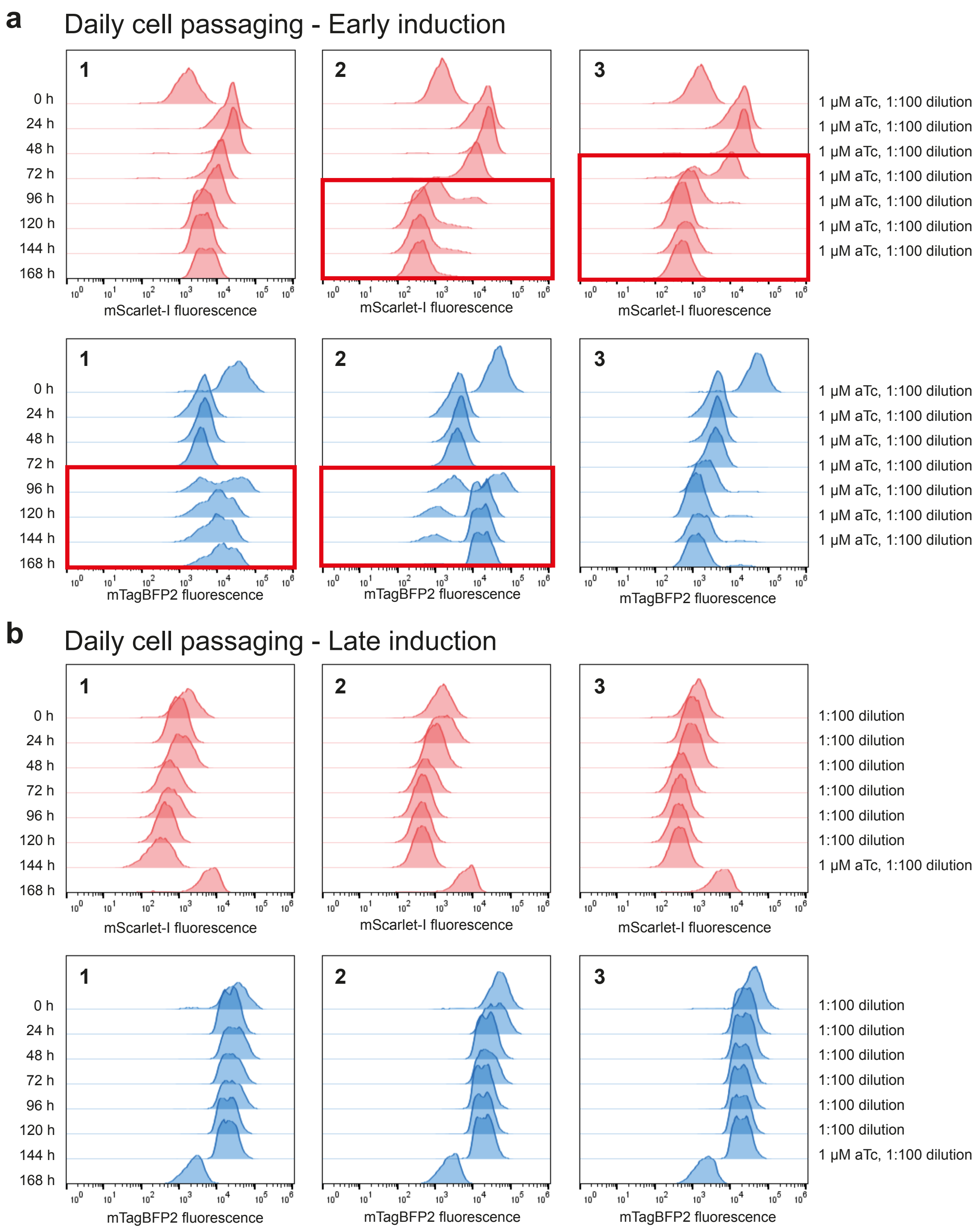

Supplementary Fig. 6. Stability of CRISPRai over 1 week of daily cell passaging. **a**, Early induction of CRISPRai. Saturated overnight cultures were diluted 1:100 into fresh SC media with 1 μM aTc at 0 h and growth at 30 °C shaking. Cells were sampled and back diluted 1:100 into fresh SC media with 1 μM aTc every 24 hours. Continually active transcription of the gRNA array starting at 0 h resulted in emergence of CRISPRai escape mutants (red box) comprising various deletions of the gRNA array, as confirmed by colony PCR (data not shown). **b**, Late induction of CRISPRai. Saturated overnight cultures were diluted 1:100 into fresh SC media without aTc and growth at 30 °C shaking. Cells were sampled and back diluted 1:100 into fresh SC media without aTc every 24 hours. At 144 h, cells were back diluted 1:100 into fresh SC media with 1 μM aTc and the final measurement was taken 24 h later. CRISPRai regulation of fluorescence performed as expected after late induction in 6 days of continuous culture, suggesting the gRNA array is stable when uninduced. Leftward drift seen in mScarlet-I fluorescence due to day variance in flow cytometer. Experimental measurements are mScarlet-I (red) and mTagBFP2 (blue) fluorescence per cell as determined by flow cytometry and shown as histograms of all gated events from individual biological replicates (> 10,000 cells).

Supplementary Table 1. Inducible CRISPRai toolkit plasmids. Plasmids names are linked to their fully annotated plasmid files on Benchling and Addgene IDs are linked to their respective Addgene page.

Name	Part Description	*E. coli* Selection	Yeast Selection	Addgene ID
**pWS3799**	Cas9 gRNA-Csy4 template	Chloramphenicol	N/A	182827
**pWS3865**	Sub-array plasmid 1/4	Ampicillin	N/A	185742
**pWS3866**	Sub-array plasmid 2/4	Ampicillin	N/A	185743
**pWS3867**	Sub-array plasmid 3/4	Ampicillin	N/A	185744
**pWS3868**	Sub-array plasmid 4/4	Ampicillin	N/A	185745
**pWS3805**	Sub-array spacer 1/4	Ampicillin	N/A	185746
**pWS3806**	Sub-array spacer 2/4	Ampicillin	N/A	185747
**pWS3807**	Sub-array spacer 3/4	Ampicillin	N/A	185748
**pWS3808**	Sub-array spacer 4/4	Ampicillin	N/A	185749
**pWS5321**	Markerless CRISPRai vector	Kanamycin	N/A	182828
**pWS5390**	*URA3* CRISPRai vector	Kanamycin	Uracil^{-}	182829
**pWS5391**	*LEU2* CRISPRai vector	Kanamycin	Leucine^{-}	182830
**pWS5392**	*HIS3* CRISPRai vector	Kanamycin	Histidine^{-}	182831
**pWS5393**	*TRP1* CRISPRai vector	Kanamycin	Tryptophan^{-}	182832
**pWS5394**	*MET17* CRISPRai vector	Kanamycin	Methionine^{-}	182833
**pWS5395**	*LYS2* CRISPRai vector	Kanamycin	Lysine^{-}	182834
**pWS5396**	KanR CRISPRai vector	Kanamycin	G418	182835
**pWS5397**	NatR CRISPRai vector	Kanamycin	Nourseothricin	182836
**pWS5398**	HygR CRISPRai vector	Kanamycin	Hygromycin	182837
**pWS5399**	ZeoR CRISPRai vector	Kanamycin	Zeocin	182838

Supplementary Table 2. gRNA targets used in this study. Repression (dCas9-Mxi1) and activation (dCpf1-VP) targets are in red and green, respectively.

Figure	Description	Target	20 bp spacer
**Fig. 1 and Supplementary Fig. 2**	Arrays contain target up to and including index number. E.g. 3x is *pALD_1*, *pALD6_2*, *pALD6_3*, *pTEF1_1*, *pTEF1_2*, *pTEF1_3*, *pHHF1_1*, *pHHF1_2*, and *pHHF1_3*.<br><br>Fig.1 is 6x in all cases.	*pALD6_1*	TATAAATGTAATAAGAAGTT
		pALD6_2	ACACCGTTCGAGGTCAAGCC
		pALD6_3	GCTGTTTGAGCTGACTAACA
		pALD6_4	TTATTACGAATTTGCCCACA
		pALD6_5	CATTGATCTCCTCTTGGGAA
		pALD6_6	AGTTAAAGCGATATAGAAGC
		pALD6_7	AAAAGAGGGTGGGCGCGCCG
		pALD6_8	TCGGCTGCCTTATTCCCGTG
		pTEF1_1	TTAACTTAAATATCAATGGG
		pTEF1_2	ACGAAGAAAAAGAAACGAGG
		pTEF1_3	GGTAATTAACGACACCCTAG
		pTEF1_4	TGAAGTGGTACGGCGATGCG
		pTEF1_5	GTAGAAACATTTTGAAGCTA
		pTEF1_6	GGGTGTGATGTAAGGATTCG
		pTEF1_7	ACATATAATACATATCACAT
		pTEF1_8	TATTATCAGCCAAAGTTGGG
		pHHF1_1	ATCATATAGAAAAAATATCT
		pHHF1_2	TTTGATGGATAAATTGGTTG
		pHHF1_3	GCAAATGCCCGCGAATACGG
		pHHF1_4	CCATTATGGGGAGAAGCGCT
		pHHF1_5	AACGCGGTTTCCAAATTCGG
		pHHF1_6	TTGTGAGAACGATAATGTAT
		pHHF1_7	TATTACGGCCAGGATCGCAA
		pHHF1_8	ACCAAAAAGAAAAATCGCCC
	Control	untargeted	GAACACTTATTCTGGGAAGA
**Fig. 2a+b**		*pRNR2*	CAAGGGTATGGTACGGTGCT
		pTEF1	TTGATATTTAAGTTAATAAA
**Fig. 3a+b, Supplementary Fig. 3, and Supplementary Fig. 4**	Fluorescent protein expression	*pRNR2_1*	CAAGGGTATGGTACGGTGCT
		pRNR2_2	TCAGCAGCAACAACACGCTA
		pRNR2_3	CCCAAACGGTTGCCCGTTGC
		pTEF1_1	TTAACTTAAATATCAATGGG
		pTEF1_2	ACGAAGAAAAAGAAACGAGG
		pTEF1_3	GGTAATTAACGACACCCTAG
	Control	untargeted	TGAGCGAATAACCTGTTGTC
		untargeted	GAACACTTATTCTGGGAAGA
**Fig. 3d-f**	Succinate (Targeted)	*ADH1*	CTTTGTATTCCAACTTACCG
		ADH3	TGTTGACGTTCTCAACATGA
		FUM1	GTGCCTGCTGATAAGTACTG
		IDP1	TGTGAGACAACAGACGCACA
		SDH1	CCGCGTCCAGATCTGCAGAC
		SDH3	CAGCTGCCTTGAGCTCCTCG
		SER3	CAACAAGCTATGAATATGAG
		SDH2	GAACGTGCTATTGAGAAGGA
		SER33	CTGAATAAGACATGTTAGGG
		ADR1	GCTATGACACCTTTTCTTTC
		ICL1	GCACCAATATTGAGTCACAT
	Succinate (Untargeted)	untargeted	TGAGCGAATAACCTGTTGTC
		untargeted	GAACACTTATTCTGGGAAGA

Gating strategy for flow cytometry

Yeast cells were gated for singlets using FSC-H vs FSC-A and to remove background noise. No other Gating was performed on global yeast population. > 10,000 events were collected and analysed within the singlets gate for each measurement.

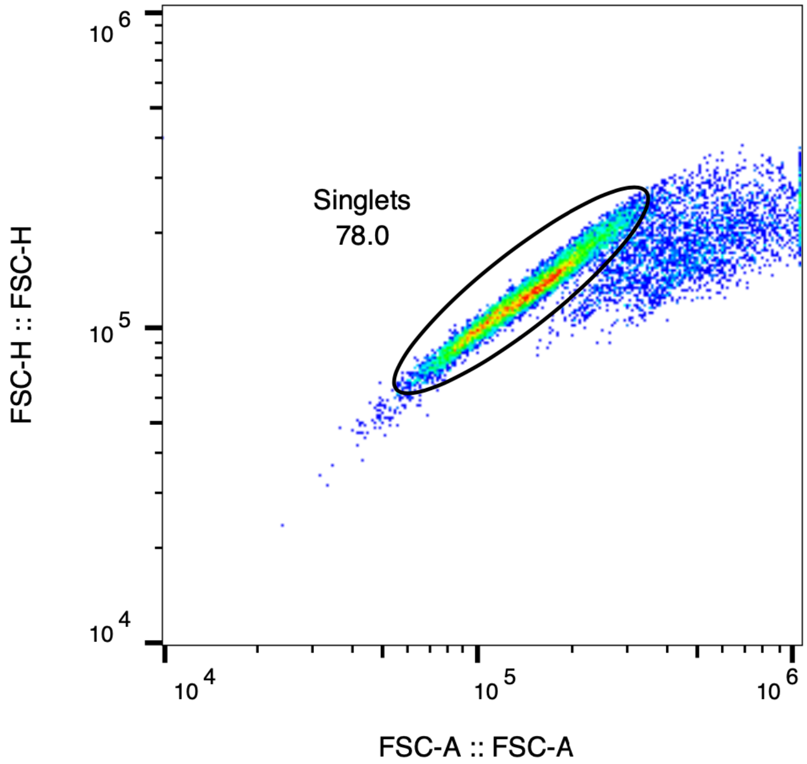

Supplement: Supplementary file 1 — Supplementary Information [file 41467_2022_32603_MOESM1_ESM.pdf]
