# Supplementary material for: Inducible expression of large gRNA arrays for multiplexed CRISPRai applications
Source: Nat Commun. 2022 Aug 25;13:4984. doi: 10.1038/s41467-022-32603-7 (PMC9411621; doi:10.1038/s41467-022-32603-7)
Supplement: Supplementary file 2 — Reporting Summary [file 41467_2022_32603_MOESM2_ESM.pdf]

nature portfolio

Corresponding author(s): Rodrigo Ledesma-Amaro
Last updated by author(s): 08/08/2022

Reporting Summary

Nature Portfolio wishes to improve the reproducibility of the work that we publish. This form provides structure for consistency and transparency in reporting. For further information on Nature Portfolio policies, see our Editorial Policies and the Editorial Policy Checklist.

Statistics

For all statistical analyses, confirm that the following items are present in the figure legend, table legend, main text, or Methods section.

n/a	Confirmed	
☐	☒	The exact sample size (*n*) for each experimental group/condition, given as a discrete number and unit of measurement
☐	☒	A statement on whether measurements were taken from distinct samples or whether the same sample was measured repeatedly
☐	☒	The statistical test(s) used AND whether they are one- or two-sided<br>*Only common tests should be described solely by name; describe more complex techniques in the Methods section.*
☒	☐	A description of all covariates tested
☒	☐	A description of any assumptions or corrections, such as tests of normality and adjustment for multiple comparisons
☐	☒	A full description of the statistical parameters including central tendency (e.g. means) or other basic estimates (e.g. regression coefficient) AND variation (e.g. standard deviation) or associated estimates of uncertainty (e.g. confidence intervals)
☐	☒	For null hypothesis testing, the test statistic (e.g. *F*, *t*, *r*) with confidence intervals, effect sizes, degrees of freedom and *P* value noted<br>*Give P values as exact values whenever suitable.*
☒	☐	For Bayesian analysis, information on the choice of priors and Markov chain Monte Carlo settings
☒	☐	For hierarchical and complex designs, identification of the appropriate level for tests and full reporting of outcomes
☒	☐	Estimates of effect sizes (e.g. Cohen's *d*, Pearson's *r*), indicating how they were calculated

Our web collection on statistics for biologists contains articles on many of the points above.

Software and code

Policy information about availability of computer code

Data collection	1. Attune NxT software v3.1 was used to collect flow cytometry data.<br>2. Molecular Devices Softmax Pro v7.1 was used to collect plate reader data.<br>3. StepOne Software v2.3 was used to collect qPCR data.<br>4. Agilent MassHunter version 10 was used to collect LC-MS data.
Data analysis	1. FlowJo v10.6.2 was used to analyse flow cytometry data.<br>2. GraphPad Prism 9 was used for generating all graphs and statistical analysis.<br>3. MicroSoft Excel was used for calculating growth rates, qPCR data, and succinic acid concentrations.<br>4. Benchling was used for designing all nucleotide sequences and CRISPR experiments.<br>5. StepOne Software v2.3 was used to analyse qPCR data.<br>6. MassHunter Quantitative software version 10 was used to analyse LC-MS data.

For manuscripts utilizing custom algorithms or software that are central to the research but not yet described in published literature, software must be made available to editors and reviewers. We strongly encourage code deposition in a community repository (e.g. GitHub). See the Nature Portfolio guidelines for submitting code & software for further information.

Data

Policy information about availability of data

All manuscripts must include a data availability statement. This statement should provide the following information, where applicable:

- Accession codes, unique identifiers, or web links for publicly available datasets
- A description of any restrictions on data availability
- For clinical datasets or third party data, please ensure that the statement adheres to our policy

Fully annotated nucleotide sequences of plasmids from the CRISPRai toolkit are available through Addgene (Addgene ID listed in Supplementary Table 1). gRNA spacer sequences for used in all experiments are available in Supplementary Table 2. All physical CRISPRai toolkit plasmids will be made available through Addgene for distribution and all additional plasmids and strains are physically available from the corresponding author upon reasonable request. All data is available from the corresponding author upon reasonable request.

Field-specific reporting

Please select the one below that is the best fit for your research. If you are not sure, read the appropriate sections before making your selection.

☒ Life sciences ☐ Behavioural & social sciences ☐ Ecological, evolutionary & environmental sciences

For a reference copy of the document with all sections, see nature.com/documents/nr-reporting-summary-flat.pdf

Life sciences study design

All studies must disclose on these points even when the disclosure is negative.

Sample size	No sample size calculation was performed. Based on previous publications (Campa et al. 2019, https://doi.org/10.1038/s41592-019-0508-6), experiments in were performed in triplicates (n = 3) or greater.
Data exclusions	No data were excluded from the manuscript.
Replication	All experiments were performed in triplicates or greater and all attempts at replication were successful (sample size indicated in figure legend).
Randomization	Transformed yeast colonies were chosen at random from plates and no data was excluded.
Blinding	The study does not contain experiments where blinding would be applicable.

Reporting for specific materials, systems and methods

We require information from authors about some types of materials, experimental systems and methods used in many studies. Here, indicate whether each material, system or method listed is relevant to your study. If you are not sure if a list item applies to your research, read the appropriate section before selecting a response.

Materials & experimental systems

n/a	Involved in the study	
☒	☐	Antibodies
☐	☒	Eukaryotic cell lines
☒	☐	Palaeontology and archaeology
☒	☐	Animals and other organisms
☒	☐	Human research participants
☒	☐	Clinical data
☒	☐	Dual use research of concern

Methods

n/a	Involved in the study	
☒	☐	ChIP-seq
☐	☒	Flow cytometry
☒	☐	MRI-based neuroimaging

Eukaryotic cell lines

Policy information about cell lines

Cell line source(s)	Saccharomyces cerevisiae Strain BY4741 from ATCC.
Authentication	We confirmed all derivative strains by PCR and sequencing.
Mycoplasma contamination	Yeast does not have this contamination.
Commonly misidentified lines (See ICLAC register)	No common misidentified lines were used.

Flow Cytometry

Plots

Confirm that:

- [x] The axis labels state the marker and fluorochrome used (e.g. CD4-FITC).
- [x] The axis scales are clearly visible. Include numbers along axes only for bottom left plot of group (a 'group' is an analysis of identical markers).
- [x] All plots are contour plots with outliers or pseudocolor plots.
- [x] A numerical value for number of cells or percentage (with statistics) is provided.

Methodology

Sample preparation	Cells were measured at approx. OD600 = 0.6 directly from SC medium with no dilution.
Instrument	Attune NxT 3 colour with Autosampler
Software	Attune NxT software for collection. FlowJo Version 10.6 for data analysis.
Cell population abundance	Typical samples included at least 10,000 cells.
Gating strategy	Yeast cells were gated for singlets using FSC-H vs FSC-A and to remove background noise. No other Gating was performed on global yeast population. > 10,000 events were collected and analysed within the singlets gate for each measurement.

- [x] Tick this box to confirm that a figure exemplifying the gating strategy is provided in the Supplementary Information.
